# Severe Work-Related Injuries in the Oil and Gas Extraction Industry — 32 Federal Occupational Safety and Health Administration Jurisdictions, United States, January 2015–July 2022

**DOI:** 10.15585/mmwr.mm7305a3

**Published:** 2024-02-08

**Authors:** Vidisha Parasram, Christina Socias-Morales, Audrey Reichard

**Affiliations:** ^1^Epidemic Intelligence Service, CDC; ^2^Division of Safety Research, National Institute for Occupational Safety and Health, CDC.

**Summary**

**What is already known about this topic?**

Oil and gas extraction (OGE) industry contract workers incur more work-related severe injuries compared with workers in other industries, based on data from the Occupational Safety and Health Administration.

**What is added by this report?**

During January 2015–July 2022, 32 jurisdictions reported 2,101 severe injuries (those resulting in amputation, loss of an eye, or inpatient hospitalization) among OGE industry workers. Overall, 895 (42.6%) reports of severe injuries involved upper extremities. Contract workers in the service and drilling subindustries experienced disproportionately more work-related injuries compared with those in the operation subindustry.

**What are the implications for public health practice?**

OGE operators could prevent contractor injuries and improve worksite safety by including contract workers in site safety management plans, improving job and equipment hazards training, and reinforcing safety practices.

## Abstract

The Occupational Safety and Health Administration (OSHA) severe injuries reports include work-related injuries from establishments under federal OSHA jurisdiction that result in an amputation, loss of an eye, or inpatient hospitalization. Data from 32 jurisdictions were examined to determine oil and gas extraction industry-specific severe industry trends during January 2015–July 2022, using the 2012 North American Industry Classification System (NAICS) codes for oil and gas extraction. During this period, a total of 2,101 severe work-related injuries were reported in this sector. Among these severe work-related injuries, well service contract workers’ injuries included the highest number of amputations (417) and hospitalizations (1,194), accounting for 20% and 57%, respectively, of all severe injuries reported. Overall, 895 (43%) of all severe injuries reported involved upper extremities. Contract workers in the service and drilling subindustries (NAICS codes 213112 and 213111, respectively) experienced disproportionately more work-related injuries compared with those in the operation subindustry (NAICS code 211). These injuries could be preventable by including contractors in worksite safety plans that administer the hierarchy of controls, are within an effective safety management system, and provide consistent safety training on work equipment, personal protective equipment, and daily site safety meetings that increase safety culture.

## Introduction

The oil and gas extraction (OGE) industry sector operates and develops oil and gas field properties. Although OGE industry sector workers represent a small portion of the U.S. workforce,* this sector is expected to grow more rapidly than other sectors.^†^ Workers in this industry are consistently overrepresented in numbers of work-related injuries, illnesses, and fatalities (*1*), possibly related to the precarious nature of their work and to their status as contract workers or self-employed.^§^

The OGE industry sector is divided into two subsectors: 1) extraction and 2) well drilling and service. The extraction subsector (North American Industry Classification System [NAICS] code 211) includes oil and gas operators, and consists primarily of companies that lease, drill, and extract fossil fuels. In contrast, the well drilling (NAICS code 213111), and service (NAICS code 213112) subsector workers are paid as contractors^¶^ who operate, construct, drill, pump, and transport oil and gas (*2*). OGE contract workers are often exposed to more hazardous work conditions (*2*) and longer shifts (*3*), and they experience more work-related fatalities (*1*,*4*). Temporary or nonstandard work arrangements have been linked to adverse health and safety outcomes, because in contrast to permanent workers, contract workers often have less information about their work environment, less job-specific training, less access to safety equipment, and no union representation (*5*,*6*). Differences have been identified within subindustries; drilling contractors experience more fatal occupational injuries and fatal falls compared with servicing employees (*4*). These risks are even higher for offshore OGE workers because of the remote, dynamic nature of platforms, and because workers live in close proximity to process units with flammable hydrocarbons (*7*).

Current data on nonfatal occupational injuries in the OGE industry sector (*8*) are limited. CDC identified risk factors for severe injuries in the OGE industry using Occupational Safety and Health Administration (OSHA) severe injury reports collected during January 2015–July 2022 to increase understanding that could guide implementation of strategies to improve OGE worker safety.

## Methods

The OSHA severe injury reports contain employer accounts of amputations, loss of an eye, or inpatient hospitalizations from 32 of 54 (59.3%) states and territories (jurisdictions) under federal OSHA authority. Severe injuries from the 22 (40.7%) jurisdictions implementing their own state-plan labor requirements are not included in the dataset. OSHA releases data from severe injury reports every 6 months.

Public OSHA severe injury reports data** collected during January 2015–July 2022 were used to examine OGE industry specific trends. Severe injury reports were aggregated by type of injury (amputation, loss of an eye, or hospitalization) and stratified by NAICS 2012^††^ and the Occupational Injury and Illness Classification System (OIICS).^§§^ Some reports included more than one injury, hospitalization, or amputation; thus, the sum of hospitalizations, amputations, and eye injuries might exceed the total number of severe injury reports. Multiple severe injures from a single report were summed to create a total number of injuries. Descriptive analyses were conducted by one- and two-digit OIICS codes for nature of injury, primary source, event or exposure, and body part affected. Descriptive analyses were stratified by time and subindustry to understand injury characteristics. Analyses were limited to cases with NAICS codes in the following subindustries: 211 (Crude Petroleum and Natural Gas Extraction), 213111 (Drilling Oil and Gas Wells), and 213112 (Support Activities for Oil and Gas Operations). Analyses were performed using SAS software (version 9.4; SAS Institute). This activity was reviewed by CDC, deemed not research, and was conducted consistent with applicable federal law and CDC policy.^¶¶^

## Results

A total of 82,366 work-related severe injuries were reported to OSHA during January 2015–July 2022; among these, 2,101 (2.6%) were reported by the OGE industry. The highest number of severe injury reports was reported by contract OGE employers. Oil and gas operations support activities personnel in well-servicing companies accounted for 1,473 (70.1%) of these 2,101 injuries, followed by oil and gas well drillers (491; 23.4%) (Table 1). Among oil and gas operators, 137 (6.5%) severe injuries were reported, including 110 (5.2%) among Crude Petroleum and Natural Gas Extraction subindustry operators and 27 (1.3%) by the Natural Gas Liquid Extraction subindustry.

**TABLE 1:** Severe injury reports* submitted to the Occupational Safety and Health Administration by oil and gas extraction industry employers, by subindustry and year (N = 2,101) — United States, January 2015–July 2022

Employer/Subindustry	Year								Total (%)
	2015	2016	2017	2018	2019	2020	2021	2022^†^	
**Contractors^§^**									
**Oil and gas subindustry support activities personnel^¶^**									
**Total severe injury reports**	**224**	**162**	**248**	**276**	**267**	**106**	**110**	**80**	**1,473 (70.1)**
Hospitalization	180	134	196	226	213	90	91	64	**1,194 (56.8)**
Amputation	59	43	72	73	81	28	36	25	**417 (19.8)**
Eye injury	2	3	3	3	2	2	3	1	**19 (0.9)**
Oil and gas well drillers**									
**Total severe injury reports**	**71**	**54**	**102**	**95**	**68**	**29**	**37**	**35**	**491 (23.4)**
Hospitalization	50	46	83	71	52	23	26	24	**375 (17.8)**
Amputation	28	10	30	40	21	7	15	12	**163 (7.8)**
Eye injury	1	0	0	0	1	0	0	0	**2 (0.1)**
**Operators^††^ in crude petroleum and natural gas extraction^§§^ and natural gas extraction^¶¶^**									
**Total severe injury reports**	**30**	**13**	**25**	**24**	**23**	**9**	**6**	**7**	**137 (6.5)**
Hospitalization	22	9	23	18	17	8	6	7	**110 (5.2)**
Amputation	10	5	6	9	8	1	1	1	**41 (2.0)**
Eye injury	0	0	0	0	1	0	0	0	**1 (0.1)**
**Total**	**325**	**67**	**375**	**395**	**358**	**144**	**153**	**122**	**2,101 (100.0)**

**Abbreviations:** NAICS = North American Industry Classification System; OSHA = Occupational Safety and Health Administration.

* Some OSHA severe injury reports included more than one injury, hospitalization, or amputation; thus, the sum of hospitalizations, amputations, and eye injuries might exceed the total number of severe injury reports. Multiple severe injures from a single report were summed to create a total number of injuries.

**^†^** During January–July 2022.

^§^ NAICS code 213.

^¶^ NAICS code 213112.

** NAICS code 213111.

^††^ NAICS code 211.

^§§ ^NAICS code 211111.

^¶¶^ NAICS code 211112.

### Temporal and Geographic Distribution of Severe Injuries

OGE severe injury reports for all subindustries fluctuated during the study period; the highest number was reported in 2018 (395), and the lowest (excluding 2022, which includes data only through July) occurred in 2020 (144). Among all severe injury reports, the highest number of amputations (417, accounting for 19.8% of reports) and hospitalizations (1,194; 56.8%) were reported among oil and gas subindustry support activities personnel in the well-servicing companies sector (163; 7.7%), followed by drilling contractors (375; 17.8%). Only 22 (1.0%) severe injury reports in OGE involved an eye injury, with oil and gas subindustry support activities personnel reporting 19 (86.4%) of these.

Among reporting jurisdictions, Texas recorded the highest number of severe injuries within OGE (1,134; 54%), followed by North Dakota (221; 10.5%) and Oklahoma (171; 8.1%). Severe injury reports occurred most frequently in July (228; 10.8%) and January (224; 10.7%).

### Body Part Involved and Nature of Severe Injuries

Analysis of injuries by involved body part found that 895 (42.6%) of all severe injury reports involved an upper extremity, 771 (86.1%) of which involved the hands; 376 (17.9%) severe injury reports involved a lower extremity, including 254 (67.6%) involving the legs (Table 2). Approximately 10% of injuries among OGE contract workers involved multiple body parts (200) or the trunk (216). In addition, contract workers in well-servicing companies recorded the highest number of hand injuries (520, accounting for 24.8% of all severe injuries) and leg injuries (183; 8.7%). Most injuries were classified as traumatic injuries and disorders (2,090; 99.5%) with open wounds (740; 35.2%), traumatic injuries to bones, nerves, and spinal cord (589; 28.0%), and other traumatic injuries and disorders (307; 14.6%) accounting for the three leading injury types. Most incidents were caused by contact with objects and equipment (1,280; 60.9%), followed by slips, trips, and falls (370; 17.6%) (Figure).

**TABLE 2:** Severe work-related injuries among oil and gas extraction workers by involved body part, nature of injury, and subindustry (N = 2,101) — United States, January 2015–July 2022

Characteristic	OIICS, no. (%)			
	Contractors		Operators^§^	Total (%)^¶^
	Support activities for oil and gas operations*	Drilling oil and gas wells^†^		
**Body part involved**				
Upper extremity**	610 (29.0)	227 (10.8)	58 (2.8)	**895 (42.6)**
Lower extremity^††^	263 (12.5)	89 (4.2)	24 (1.1)	**376 (17.9)**
Multiple body parts^§§^	156 (7.4)	44 (2.1)	16 (0.8)	**216 (10.3)**
Trunk^¶¶^	156 (7.4)	47 (2.2)	8 (0.4)	**211 (10.0)**
Head***	114 (5.4)	40 (1.9)	11 (0.5)	**165 (7.9)**
Nonclassifiable^†††^	85 (4.0)	19 (0.9)	12 (0.6)	**116 (5.5)**
Body systems^§§§^	81 (3.9)	23 (1.1)	7 (0.3)	**111 (5.3)**
Neck, including throat^¶¶¶^	6 (0.3)	2 (0.1)	0 (—)	**8 (0.4)**
**Total**	**1,471 (70.0)**	**491 (23.4)**	**136 (6.5)**	2,098 (99.9)****
**Nature of injury**				
Open wound	508 (24.2)	186 (8.9)	46 (2.2)	**740 (35.2)**
Traumatic injury to bones, nerves, or spinal cord	425 (20.2)	133 (6.3)	31 (1.5)	**589 (28.0)**
Other traumatic injury or disorder	205 (9.6)	78 (3.7)	24 (1.1)	**307 (14.6)**
Burn or corrosion	148 (7.0)	29 (1.4)	21 (1.0)	**198 (9.4)**
Multiple traumatic injuries or disorders	57 (2.7)	20 (1.0)	0 (—)	**77 (3.7)**
Effect of environmental conditions	37 (1.8)	13 (0.6)	2 (0.1)	**52 (2.5)**
Traumatic injury or disorder, unspecified	29 (1.4)	7 (0.3)	4 (0.2)	**40 (1.9)**
Intracranial injury	27 (1.3)	12 (0.6)	3 (0.1)	**42 (2.0)**
Traumatic injury to muscles, tendons, ligaments, or joints	18 (0.9)	7 (0.3)	2 (0.1)	**27 (1.3)**
Surface wound or bruise	11 (0.5)	4 (0.2)	2 (0.1)	**17 (0.8)**
**Total**	**1,465 (69.7)**	**489 (23.3)**	**135 (6.4)**	**2,089 (99.4)^†††^**

**Source**: OIICS Code Trees. https://wwwn.cdc.gov/wisards/oiics/Trees/MultiTree.aspx?TreeType

**Abbreviations:** NAICS = North American Industry Classification System; OIICS = Occupational Injury and Illness Classification System.

* NAICS code 213112.

^†^ NAICS code 213111.

^§^ NAICS code 211.

^¶^ Total percentage row totals might not equal the sum of all percentage values in a row because of rounding.

** Includes the extremities that are bounded by the trunk at the top with the fingers as the lowermost part (e.g., bones, cartilage, muscles, skin, subcutaneous tissue, veins, and arteries of upper extremities).

^††^ Includes the appendages that are bounded by the hip to the top with the toes as the lowermost part (e.g., bones, cartilage, muscles, skin, subcutaneous tissue, veins, and arteries of lower extremities).

^§§^ Multiple body parts from two or more areas of the body.

^¶¶^ The main part of the body where the head and limbs are attached. The area is bounded by the neck, shoulders, and legs.

*** The uppermost parts of the body (e.g., the skull, its contents, and related external structures; excludes amputations).

^†††^ Source not known.

^§§§^ The functioning of an entire body system is affected without specific injury (e.g., hypothermia or asthma).

^¶¶¶^ The portion of the body that connects the head to the torso or trunk (e.g., the jaw, chin, and cranial region to the top and the shoulder below; excludes amputations).

**** Data were missing for three severe work-related injury records.

**FIGURE:**
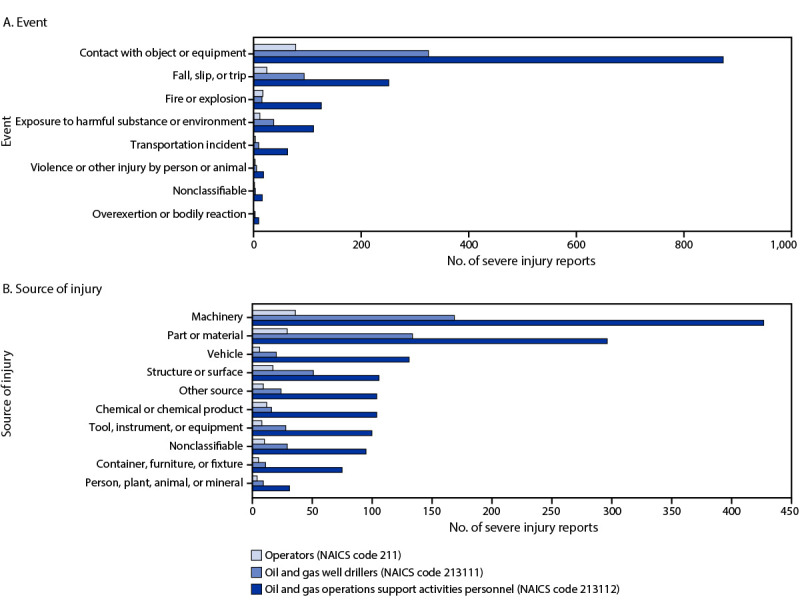
Severe work-related injuries* among oil and gas extraction workers, by event (A) and source of injury (B) (N = 2,101) — United States, January 2015–July 2022 **Abbreviation:** NAICS = North American Industry Classification System. * Injuries that result in an amputation, loss of an eye, or inpatient hospitalization.

### Sources of Severe Injuries

Machinery was the leading source of injury (633; 30.1%) among OGE contractors and operators, with construction, logging, and mining machinery accounting for 483 of these injuries (23.0% of all injuries). The second most common cause of injury involved parts and materials (460; 21.9%), followed by structures and surfaces (174; 8.3%). Among these, building materials-solid elements (187; 8.9%) was the leading source of injury. Vehicles*** were involved in 157 (7.5%) severe injuries and were the third highest source of injury among contractors in well-servicing companies, accounting for 131 (6.2%) injuries among these groups. Highway motorized vehicles^†††^ (e.g., passenger vehicles, trucks, and multipurpose vehicles) accounted for 101 (4.8%) severe injuries. Overall, 430 (20.5%) injuries involved oil drilling rigs and machinery, and several involved other equipment, including pipes, ducts, and tubing (101; 4.8%), machine and appliance parts (67; 3.2%), heat-environmental equipment (52; 2.5%), and hoses (37; 1.8%). 

## Discussion

Although OGE workers represent a small proportion of the U.S. workforce, these workers are consistently overrepresented in reports of work-related injuries, illnesses, and fatalities (*1*). Among OGE workers, contract workers in oil and gas subindustry support activities personnel in the well-servicing subindustry experience a greater number of severe work-related injuries than do those in the drilling contractor and operator subindustries. This finding might be attributed to the temporary nature of most work in this subindustry, which is largely without a social safety net, and consists of high-hazard jobs for which workers do not receive consistent training (*6*). Most of these severe injuries affect the upper and lower extremities, involve machinery or parts and materials, and vehicles, and are caused by contact with objects or trips, slips, and falls. These severe injuries might be associated with work stress, exposures to hazardous chemicals and other comorbid conditions, and vulnerabilities that are not available in the severe injury report data for analysis but warrant further research.

Under OSHA’s General Duty Clause,^§§§^ an employer must ensure a safe workplace for employees. This responsibility is allocated to OGE operators, who hire site contractors with their own safety programs that might not address all the site and equipment hazards present at a worksite. One potential strategy to address this would be for OGE operators to involve workers and contractors with a thorough understanding of work conditions in creating a job hazard analysis or daily safety plan within an effective safety management system. Using a safety management system that employs stringent and consistent safety training on job equipment, including personal protective equipment, and incorporates daily site safety meetings to discuss and address the changing work hazards can foster an inclusive safety culture. Further, severe injuries could be prevented by employing a hierarchy of controls, a process for identifying and controlling hazards^¶¶¶^ whereby the most effective controls involve eliminating or substituting the hazard or condition through engineering controls, followed by safe work practices, administrative controls, and use of personal protective equipment when feasible.****

### Limitations

The findings in this report are subject to at least five limitations. First, severe injury reports are administrative records collected for enforcement rather than a census or sample of work-related injuries for public health research; these data lack information on individual workers and are only available at the facility level, thereby limiting analysis. Second, only those severe injury reports from federal jurisdictions are publicly available. States implementing their own state plans are subject to the same reporting requirements, however, these data are not publicly available; thus, data from states with a large oil and gas sector (e.g., California, New Mexico, Utah, and Wyoming) are not available for analysis, limiting understanding of severe injury trends nationally in the OGE sector. Third, despite the reporting requirement, injuries are significantly underreported to OSHA.^††††^ Fourth, the data do not contain worker demographic and work arrangement information that would permit identification of high-risk worker populations or health and safety inequities. Finally, the data do not contain information on injury severity or length of hospital stay, thereby limiting analysis of risk.

### Implications for Public Health Practice

OSHA severe injury reports data provide timely, transparent, publicly available injury information at no cost to users, which can be used to examine trends over time, by geographic region, and by injury characteristics. These data have previously been analyzed to examine kidney injuries among indoor and outdoor workers (*9*) and seasonality and trends (*10*). The current severe injury report is the first of its kind to record nonfatal severe occupational injuries among federally covered states. These data can increase awareness of nonfatal injuries in the OGE sector on a national level by describing (through the use of NAICS and OIICS codes) the industry, nature, primary source, event of the injury, and affected body part or parts when severe occupational injuries occur. OSHA severe injury reports are submitted by employers but are confirmed and coded by OSHA. Despite significant underreporting, they provide additional insight into the occurrence of severe occupational injuries and can therefore guide the development of strategic interventions for severe injury prevention in the OGE industry. The data also provide an opportunity to monitor changes in occupational injury trends in 6-month increments instead of annual data releases available from other occupational injury surveys. These findings also underscore the necessity for OGE operators to work with contracting companies to review their health and safety programs, interventions, and company safety procedures and address specific worksite hazards to prevent the occurrence of severe injuries leading to hospitalizations and amputations specifically affecting upper and lower body extremities.

## Footnotes

*
https://www.bls.gov/iag/tgs/iag21.htm

†
https://www.eia.gov/outlooks/aeo/

§ The OGE industry largely consists of a contract or self-employed workforce.

¶
https://www.cdc.gov/niosh/docs/2017-193/2017-193.pdf

**
https://www.osha.gov/severeinjury

††
https://www.census.gov/naics/?58967?yearbck=2012

§§ Version 2.01. https://wwwn.cdc.gov/wisards/oiics

¶¶ 45 C.F.R. part 46; 21 C.F.R. part 56; 42 U.S.C. Sect. 241(d), 5 U.S.C. Sect. 552a, 44 U.S.C. Sect. 3501 et seq.

*** Total number of vehicles involved is determined from the OIICS code under source of injury or illness and defined as vehicles that generally move on wheels, runners, water, or air.

††† Motorized vehicles are vehicles which are operated primarily on highways and other public roadways and used for transportation, hauling, delivering, and emergencies.

^ §§§^ https://www.osha.gov/laws-regs/oshact/section5-duties

¶¶¶
https://www.cdc.gov/niosh/topics/hierarchy/default.html

****
https://www.osha.gov/safety-management/hazard-prevention

††††
https://www.osha.gov/sites/default/files/severe-injury-report-2015to2021.pdf
